# Advancements in Diagnostics and Therapeutics for Cancer of Unknown Primary in the Era of Precision Medicine

**DOI:** 10.1002/mco2.70161

**Published:** 2025-04-16

**Authors:** Ting Zhao, Xiaowei Zhang, Xin Liu, Qifeng Wang, Xichun Hu, Zhiguo Luo

**Affiliations:** ^1^ Department of Medical Oncology Fudan University Shanghai Cancer Center Shanghai China; ^2^ Department of Oncology Shanghai Medical College Fudan University Shanghai China; ^3^ Department of Pathology Fudan University Shanghai Cancer Center Shanghai China

**Keywords:** cancer of unknown primary, molecular diagnostics, empirical chemotherapy, site‐specific therapy, molecularly guided therapy

## Abstract

Cancer of unknown primary (CUP), a set of histologically confirmed metastases that cannot be identified or traced back to its primary despite comprehensive investigations, accounts for 2–5% of all malignancies. CUP is the fourth leading cause of cancer‐related deaths worldwide, with a median overall survival (OS) of 3–16 months. CUP has long been challenging to diagnose principally due to the occult properties of primary site. In the current era of molecular diagnostics, advancements in methodologies based on cytology, histology, gene expression profiling (GEP), and genomic and epigenomic analysis have greatly improved the diagnostic accuracy of CUP, surpassing 90%. Our center conducted the world's first phase III trial and demonstrated improved progression‐free survival and favorable OS by GEP‐guided site‐specific treatment of CUP, setting the foundation of site‐specific treatment in first‐line management for CUP. In this review, we detailed the epidemiology, etiology, pathogenesis, as well as the histologic, genetic, and clinical characteristics of CUP. We also provided an overview of the advancements in the diagnostics and therapeutics of CUP over the past 50 years. Moving forward, we propose optimizing diagnostic modalities and exploring further‐line treatment regimens as two focus areas for future studies on CUP.

## Graphical abstract

In clinical practice, routine examinations for patients with CUP include taking a medical history, conducting a physical examination, and detecting serum tumor markers. In addition, IHC or MTP‐based methodologies were suggested to assist in identifying the tissue origin of CUP and guiding site‐specific therapy for CUP.

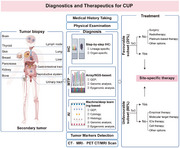

## 1 Introduction

Cancer of unknown primary (CUP) refers to a group of histologically confirmed metastatic malignancies that cannot be identified or traced back to its primary site despite comprehensive examination, which accounts for 2–5% of all tumors [1, 2, 3, 4, 5, 6, 7]. CUP ranks as the seventh to eighth most common type of malignancy and is the fourth leading cause of cancer‐related deaths worldwide [6, 8]. Despite recent therapeutic advancements, patients with CUP still face a bleak prognosis, with a median overall survival (OS) of 3–16 months [6, 9, 10], which is primarily attributed to the diagnostic challenges for CUP. Actually, patients with CUP are categorized into favorable (15–20%) and unfavorable (80–85%) subgroups with distinct prognosis, according to their clinical and pathological characteristics [1]. Recently, new favorable subsets of CUP seem to emerge, including colorectal, lung, and renal CUP [11]. In fact, fewer than 30% of cases of CUP have specified lesions identified prior to systemic therapy before 2000.

CUP is characterized by high heterogeneity, early dissemination, strong invasiveness, and unpredictable metastasis patterns. Incidence of CUP varies across countries due to the absence of a precise definition and differences at the health facility level. On the whole, CUP typically occurs around the age of 60 years, with rare cases observed in individuals under 40 years of age and a peak incidence in patients over 80 years of age. Males consistently have a slightly higher incidence compared with females [2]. In addition to age and sex, other potential risk factors include long‐term smoking, alcohol abuse, autoimmune disorders, diabetes mellitus, and a family history of cancer [12, 13, 14, 15, 16, 17]. However, there is still a lack of sufficient epidemiological evidence to determine a distinct risk factor profile for CUP.

Identifying the primary site for CUP remains challenging due to several reasons, principally including incomplete examination, insufficient pathological sampling, unique tumor metastatic patterns, occult primary sites, spontaneous regression of the primary tumor, and absence of primary lesions. Over the past 50 years, significant efforts have been dedicated to identifying the tissue origin of CUP in order to optimize therapeutic strategies. In addition to comprehensive history taking and full physical examination, a variety of diagnostic techniques, such as imaging and pathological examination, gene expression profiling (GEP), as well as genomic and epigenomic analysis, have been applied to diagnose CUP [8, 18, 19]. Histopathological analysis, including morphological analysis and immunohistochemistry (IHC) on specimens, was the most commonly used methodology for diagnosing CUP in early‐stage [20, 21, 22, 23]. Currently, a step‐by‐step IHC staining with lineage‐ and organ‐specific markers is adopted to assist in identifying the tissue origin of CUP [23, 24], which enables the development of individualized therapeutic options tailored to the specific organ or histological type. We have to admit, however, the IHC staining also has many limitations, particularly in poorly differentiated and undifferentiated tumors. In this case, the diagnostic accuracy of IHC may be compromised, which poses challenges in accurately determining the tumor tissue origin [10]. Published studies revealed that the overall diagnostic accuracy of IHC alone for CUP is approximately 77% [25]. Over the past two decades, there have been significant advancements in molecular diagnostics, making the widespread adoption and recognition of GEP and next‐generation sequencing (NGS) as two effective methodologies for the diagnosis of CUP [26, 27, 28]. These methodologies have demonstrated superiority over IHC, with an accuracy of 83–94% [29, 30, 31]. However, the molecular tumor profiling (MTP) is not always performed and still lacks clinical penetration in resource‐poor settings. With the advancements of artificial intelligence (AI) in clinical diagnosis of certain tumor types, machine learning and deep learning algorithms based on cytology, histology, GEP, and NGS‐based genomic and epigenomic analysis have been extensively validated to improve the diagnostic accuracy of CUP [32, 33, 34, 35, 36], which opens a fresh chapter in the diagnosis and management for patients with CUP.

Challenges in diagnosis present significant obstacles to clinical therapy for CUP, further contributing to a worse prognosis for CUP. In the past five decades, empirical chemotherapy has been widely acknowledged as the cornerstone for the treatment of CUP. Organ‐specific therapy, tailored to the identified tumor tissue origin, has been proven to be effective. However, in subsets of CUP with undefined tumor tissue origin, empirical treatment using combination regimens of taxane or platinum is employed, leading to less optimal patient outcomes [10]. To further improve the prognosis of patients with CUP, several clinical trials have been conducted to explore the benefits of MTP‐directed site‐specific first‐line therapy over empirical chemotherapy [37, 38, 39, 40, 41, 42]. In 2013, the Sarah Cannon Research Institute conducted the first prospective trial worldwide to indicate that patients with CUP who received GEP‐directed first‐line site‐specific treatment exhibited improved OS compared with those who received empirical therapy [37]. However, another phase II clinical trial performed by Kindai University Faculty of Medicine reported that GEP‐directed site‐specific treatment did not yield a significant improvement in OS and progression‐free survival (PFS) compared with empirical chemotherapy [38]. Furthermore, a recent study from Moffitt Cancer Center demonstrated that patients who underwent NGS‐directed site‐specific therapy had a longer OS compared with those treated with standard options, although this comparison did not reach statistical significance [40]. However, neither of these studies has confirmed the superiority of site‐specific therapy over empirical chemotherapy, which may be attributed to the significant deficiencies present in the currently available studies. These limitations include patient recruitment (oversampling treatment‐resistant tumor types and long recruitment), study design limitations (retrospective and observational trials), heterogeneity among the classifiers (different omic approaches with variable performance), as well as incomparable treatment regimens [43]. In light of this, our center conducted the world's first phase III clinical trial and demonstrated improved PFS and favorable OS with GEP‐guided site‐specific therapy for patients with CUP [41, 42], which provides new strategies for the treatment and clinical management for CUP in the future.

In this review, we have detailed the epidemiology, etiology, pathogenesis, and histologic, genetic, and clinical features of CUP, as well as provide an overview of the advancements in the clinical diagnosis and treatment of CUP. We have proposed and established the status of site‐specific first‐line therapy in improving patient outcomes of CUP through the world's first phase III clinical trial. Meanwhile, we are now working on exploring effective second‐line therapy regimen for previously treated or progressed CUP, and the data will be made public in the near future. Furthermore, we construct a hierarchical management system for the diagnosis, treatment and follow‐up of CUP. Looking ahead, we believe that the optimization of diagnostic approaches and the exploration of second‐line regimens are two crucial areas in future studies for CUP, which still have a long way to go.

## 2 Epidemiology of Cup

Over the past few decades, the incidence rate of CUP has varied between developed and developing countries, with an overall global incidence ranging from 2 to 5% from a holistic perspective [44]. Overall, geographic differences in the morbidity of CUP were attributed to the absence of a unified criterion for defining and diagnosing CUP. Between 1961 and 2010, the age‐standardized incidence rate (ASIR) of CUP was about 5–18 per 100,000 in Scotland, reaching a peak in the early to middle 1990s followed by a steeper decrease, accounting for 3.9% of all registrations of tumors [45]. Meanwhile, from 1960 to 2008 in Sweden, the ASIR of patients with CUP was approximately four to eight per 100,000, exhibiting a peak in rates in the late 1990s followed by a significant decrease, and accounted for 3.9% of all diagnosed cancers [46]. In Switzerland, the ASIR of CUP increased from 10.3 to 17.6 per 100,000 between 1981 and 1997, and then decreased to 5.8 in 2014, representing approximately 0.9–2.6% of newly diagnosed cancers [4]. Based on epidemiological statistics from Europe, we observed a consistent trend of peak of ASIR in the 1990s followed by an obvious decline, which may primarily result from constantly improved diagnostic methods to identify the primary sites and decreased autopsy rates. Compared with data from Europe, the USA experienced earlier decreases in ASIR since the early 1980s. Between 2000 and 2010 in the USA, the average ASIR of CUP was 4.1 per 100,000 [47]. Until now, however, there has been no detailed epidemiological report of the incidence rate of CUP in the Asian population. According to the latest statistics from Fudan University Shanghai Cancer Center, which analyzed 1420 pathologically confirmed CUP between 2019 and 2020, CUP accounted for approximately 0.82% of all cancer diagnoses [48]. Actually, the observed relatively low and significant decrease of incidence rate in CUP may primarily be due to consistent advancements in diagnostic techniques [5].

## 3 Etiology and Pathogenesis of Cup

In terms of etiology, previous studies have indicated that human papillomavirus is a definite causative factor in squamous cell carcinomas arise in the head and neck, abdomen, pelvis, and retroperitoneum with unknown primary tumor sites [49, 50, 51]. Furthermore, long‐term smoking, alcohol abuse, diabetes mellitus, obesity, autoimmune disorders, and a family history of cancer have been suggested as potential risk factors for CUP [13, 14, 16, 52, 53]. In addition, age and gender were another two established risk factors for CUP. Patients with CUP typically experience a peak incidence at the age of 80 years, with the average onset of the disease occurring around the age of 60 years. Further, the incidence of CUP progressively increases with advancing age [54]. Male gender is also considered as a potential risk factor for patients with CUP [2]. In addition, black ethnic background and low socioeconomic status may be considered as other possible risk factors for CUP [52, 55]. However, no significant correlation has been observed between dietary habits, lifestyle, and physical activity levels and onset of CUP [13, 56, 57]. Overall, no specific causative factors or risk factors have been confirmed for CUP that differ significantly from those associated with other types of metastases with known primaries.

**FIGURE 1:**
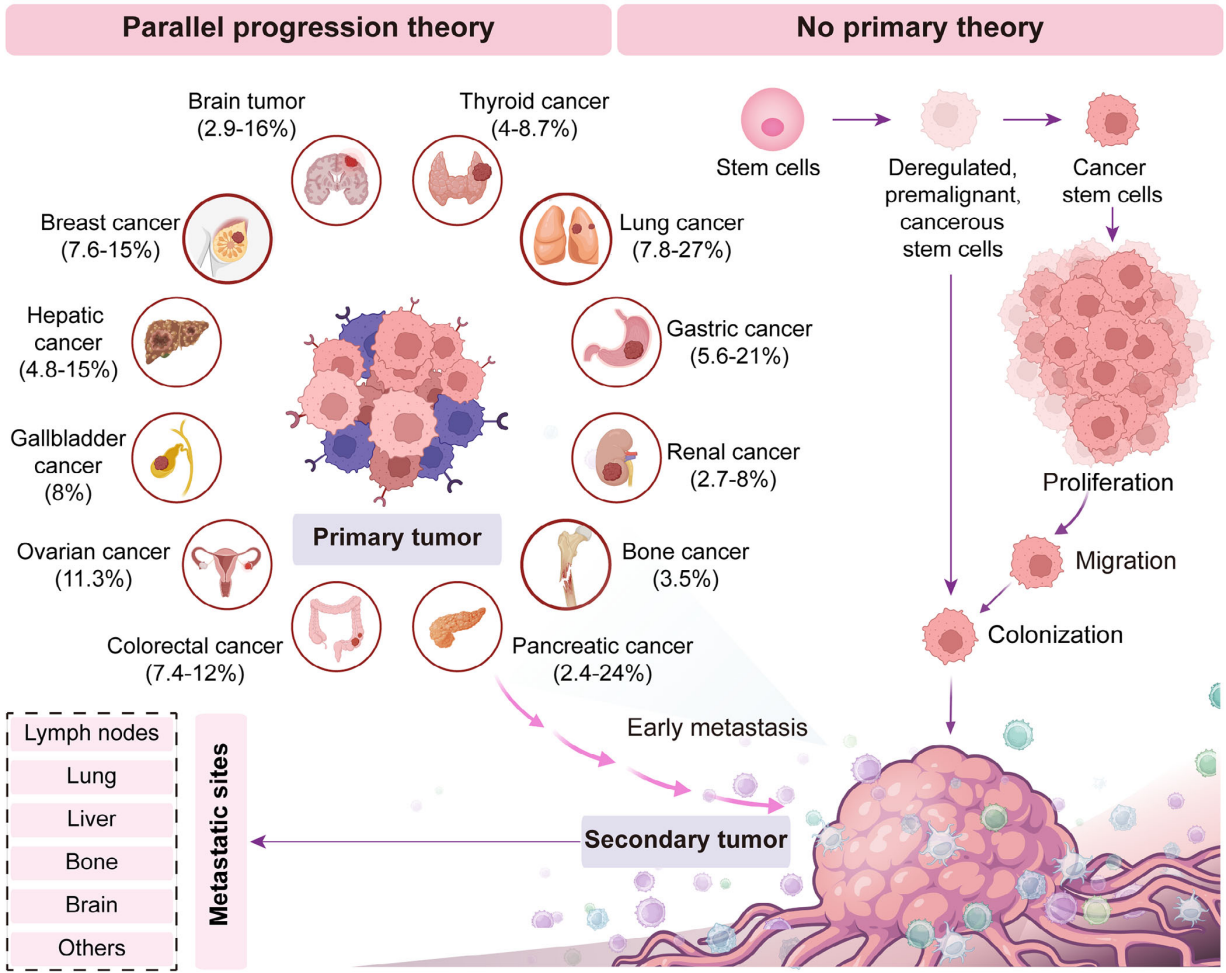
The underlying mechanisms involved in the carcinogenesis and progression of CUP. In the first scenario, the “no primary theory” suggests that deregulated, premalignant, or cancerous stem cells may migrate to a distant site and give rise to a new neoplasm, without forming a tumor at the primary site. While in the second scenario, the “parallel progression theory” agrees that mobile cells migrate to metastatic sites at an early stage and suppress the development of the primary tumor, leading to undetectable local lesions. The percentage in the figure represents the probability of the primary tumors occurring among all cases of CUP, and the graphic was created with BioRender.

Since the occult of primary site and the heterogeneity of metastases, there is no define consensus on the pathogenesis of CUP. Currently, there have been two main theories regarding the pathogenesis of CUP. In the first scenario, deregulated, premalignant, or cancerous stem cells migrate to a distant site and initiate a cancer, without forming a tumor at the primary site [58]. Alternatively, the primary lesions disappear before they develop into detectable lesions [59, 60]. In this case, the primary tumor never exists, which is known as “no primary theory.” This may explain why many CUP patients in clinical settings, despite undergoing various diagnostic procedures, are still unable to trace the primary tumor site. In the second scenario, mobile cells migrate to metastatic sites at an early stage and reprogram the tumor microenvironment to enhance the growth of metastasis [60]. Further, distant metastasis inhibits the development of local growth [58, 61] and finally contributes to the occult capacity of the primary tumors, which lays the foundation for the identification of the primary tumor site and site‐specific therapy. In this situation, approximately 4–25% of cases with CUP finally identify the primary lesions during the course of disease [62], which was acknowledged as “parallel progression theory” [60] (Figure 1). However, it is important to note that the aforementioned theories have not been confirmed and still need further exploration and validation.

## 4 Histologic, Genetic, and Clinical Features of Cup

According to the morphological features, CUP can be categorized into four histological types: well and moderately differentiated adenocarcinomas (50%), undifferentiated or poorly differentiated adenocarcinomas (30%), squamous cell carcinomas (15%), and undifferentiated neoplasms (5%) [4, 8, 63]. Sarcomas, melanomas, neuroendocrine tumors, germ cell tumors, and hematological malignancies with unknown primary sites are excluded from the definition of CUP. One particular concern is approximately 3% of melanomas lack an identifiable primary were defined as melanoma of unknown primary, which seems to present better outcomes compared with those with stage‐matched melanoma of known primary [64]. Actually, different histological subtypes of CUP exhibit varying responses to treatment. Survival is worse for patients with adenocarcinoma and undifferentiated carcinoma in comparison with those with squamous cell carcinomas (1‐year survival rate of 19 and 46%, respectively) [55], which is primarily due to the varied genetic characteristics and different activated oncogenic signaling pathway among distinct histological types of CUP.

From genetic perspective, the most frequent genomic alterations observed were *TP53*, *KRAS*, *CDKN2A*, *MET*, *MYC, ERBB2, PIK3CA, FGFR2, BRAF*, and *ARID1A* [19, 65‐70]. Among these, approximately half of the patients with CUP experienced *TP53* mutation, while *CDKN2A* was the most frequently deleted gene in CUP [71]. In addition, alterations of *ERBB2*, *EGFR* and *BRAF* are more common in adenocarcinoma of unknown primary (ACUP) compared with non‐ACUP, whereas alteration of *MLL2* is more frequent in non‐ACUP [19]. In cases of CUP, the most frequently observed oncogenic gene partner is *FGFR2*, followed by ALK [71], providing evidence for targeted therapy. Usually, the intact kinase domain of *FGFR2* forms a fusion with another gene, which provides a dimerization domain to activate RAS/MAPK and PI3K/AKT downstream pathways. Previous study demonstrated that ACUP showed elevated activation of the RAS/MAPK signaling pathway in comparison with those with non‐ACUP [19], which may be attributed to the increased expression of *FGFR2*, *KRAS*, and *ALK* in cases of ACUP. Further, high expression of PD‐L1 (TPS > 50%) has been documented to be present in approximately 14% patients of CUP [65], with the positivity rate showing no significant differences among different histological subtypes [72]. Meanwhile, about 16% of CUP exhibited high tumor mutational burden (TMB) (>10 mutations/Mb) [73]. In fact, previously published studies documented that about 28% of patients with CUP harbor one or more predictive biomarkers (PD‐L1, microsatellite instability [MSI]‐high, and/or TMB‐high) for immune checkpoint inhibitors (ICIs) treatment [74]. PD‐L1 expression, MSI, and TMB status are acknowledged as predictive biomarkers when ICIs application is considered. Furthermore, chromosomal instability (CIN) is not frequently observed in CUP [12, 75]. CIN is considered as a driver of immunosuppression and cancer metastasis [76, 77, 78], which may make ICIs more beneficial for individuals with CUP. Recently, Professor Mileshkin applied immunotherapy response (IR) score (defined according to specific immune gene‐expression profiles), TMB status, and ICI‐responsive cancer types as predominant biomarkers to predict the ICIs response in CUP, achieving an overall response rate (ORR) of 29% [73]. IR score, in particular, was more sensitive than TMB and cancer types to predict response to ICIs. Furthermore, HER‐2 was found to be overexpressed in approximately 10% of CUP [27, 79], which provides opportunities for HER2‐targeting antibody–drug conjugate (ADC) treatment for patients with CUP. From the protein level, cell cycle proteins (TP53, BCL2) [80], oncoproteins (MYC, RAS) [81], and matrix metalloproteinases (MMP‐2, MMP‐9, and TIMP‐1) [82] were found to be highly expressed in patients with CUP, which may contribute to the development and progression of CUP.

For CUP, the average onset of the disease is around the age of 60 years [48, 54], with a higher incidence observed in males compared with females. Patients with CUP often present to the clinic for symptoms and signs of metastatic tumors, including lymphadenopathy, local mass, bloating, pain, jaundice, weight loss, or hoarseness [83]. Among these, lymph nodes are the most commonly involved lesions of metastasis, accounting for approximately 14–41.8% of all cases [48, 84, 85]. As a result, lymphadenopathy plays a crucial role in tracing the primary lesions of CUP, and our group was the first to propose the concept of the “sentinel node theory” to tract the primary for patients with CUP [86, 87]. Lung [85, 88], gastrointestinal tract [37], liver [39], pancreas [89], and breast [30] were predicted as the most frequently identified primary sites for CUP, providing a solid foundation for site‐specific therapy. In addition, patients with CUP were divided into favorable (15–20%) and unfavorable (80–85%) subsets according to the clinical and pathologic characteristics of CUP [1]. Patients with CUP in the favorable subgroup include women with papillary adenocarcinoma of the peritoneal cavity, women with adenocarcinoma involving the axillary lymph nodes, poorly differentiated carcinoma with midline distribution, squamous‐cell carcinoma involving cervical lymph nodes, men with blastic bone metastases and elevated prostate‐specific antigen (PSA), isolated inguinal adenopathy (squamous carcinoma), adenocarcinoma with a colon‐cancer profile (CK20+, CK7−, CDX2+) (adenocarcinoma), and patients with one small, potentially resectable tumor. Recently, new favorable subsets of CUP seem to emerge, which include colorectal, lung, and renal CUP [11]. However, patients with multiple cerebral metastases (adenocarcinoma or squamous carcinoma), adenocarcinoma metastatic to the liver or other organs, multiple metastatic lytic bone disease (adenocarcinoma), pleural metastases (adenocarcinoma), squamous‐cell carcinoma of the abdominopelvic cavity, and nonpapillary malignant ascites (adenocarcinoma) belong to the prognostically unfavorable subset [1, 9].

## 5 Diagnosis of Patients With Cup

### 5.1 Medical history taking and physical examination

Medical history taking includes a thorough evaluation of symptoms and signs, previous examinations, preexisting conditions, medical and surgical history, and the family history of cancer. Specialist physical examinations should involve the assessment of local masses, superficial lymph nodes, breast, urogenital tract, and rectum to provide clues for identifying the primary lesions for CUP. In cases with CUP, lymphadenopathy is the most commonly observed and crucial positive sign [90]. Cancer cells frequently disseminate through the lymphatic system to initiate secondary tumors in distant sites, separate from the primary tumor. The drainage pathway for tumor typically follows a predictable pattern throughout the body, providing valuable clues for tracking the primary tumor site for CUP. For instance, cervical lymphadenopathy often indicates head and neck squamous carcinoma, as well as metastases from salivary gland, thyroid, lung, breast, gastrointestinal, and genitourinary tract tumors. In fact, a previous study conducted by our center confirmed that the predictive accuracy of “sentinel node theory,” particularly for differentiated carcinoma, was 95% in tracking the primary tumors [86].

### 5.2 Serum tumor markers detection

Since their discovery in 1978, serum tumor markers have been widely utilized in clinical settings for tumor screening, diagnosing, recurrence monitoring, and treatment efficacy assessing. In certain cases, the expression level of specific tumor markers, such as lactate dehydrogenase and carcinoembryonic antigen, may have prognostic significance for survival [91]. However, tumor markers do not provide adequate diagnostic assistance for patients with CUP due to their limited sensitivity and specificity. Indeed, tumor markers were often detected as a reference indicator alongside other examinations including pathology and imaging. In some certain types of cancer, specific tumor markers, for instance, alpha‐fetoprotein in hepatic carcinoma and PSA in prostate cancer, have shown high sensitivity and specificity for specific tumor types, which provides valuable clues for identifying the primary site for CUP. In clinical practice, continuous monitoring of tumor markers is performed to assess the efficacy of anticancer therapy and to monitor tumor recurrence for CUP.

### 5.3 Positron emission tomography scan

Contrast‐enhanced computed tomography (CT) and magnetic resonance imaging (MRI) are commonly used in the routine diagnosis of tumors. Since the great advancements of molecular imaging in recent years, positron emission tomography (PET), combined with CT or MRI, has emerged as a widely used approach for diagnosing and staging tumors. ^18^F‐fluorodeoxyglucose (FDG) PET/CT has been applied extensively in the diagnosis, therapy monitoring, and prognostic assessment in patients with CUP [92, 93, 94, 95, 96, 97, 98]. For patients presenting with lymph node metastases of unknown primary sites, the overall sensitivity, specificity, and accuracy rates of ^18^F‐FDG PET/CT were 73, 89, and 81%, respectively [92]. In particular, for patients with cervical lymph node metastases of unknown primary, ^18^F‐FDG PET/CT illustrated a higher overall sensitivity and specificity of 88.3 and 74.9%, along with an accuracy of 78.8%, respectively. Meanwhile, another retrospective study involving 31 CUP patients with cervical lymph node metastasis suggested that the sensitivity and specificity of ^18^F‐FDG PET/CT in detecting the primary site were 67 and 91%, respectively [95]. A point worth emphasizing is that in approximately 25% of patients with CUP, ^18^F‐FDG PET/CT accurately identified the primary lesions that had gone undetected by other procedures. And, it revealed previously unrecognized regional or distant metastases in 27% of patients [94]. SUV_max_ on pretreatment ^18^FDG‐PET/CT scanning has been accepted as a prognostic indicator in certain cancer types, such as and non‐small‐cell lung cancer (NSCLC) [99], salivary gland carcinoma [100], and epithelial ovarian cancer [101]. In patients with CUP, an SUV_max_ above 20 on ^18^F‐FDG PET/CT has been proven to be associated with a favorable OS [93], which may be attributed to the predictive value of SUV_max_ in determining the tumor chemosensitivity. In addition, ^68^Ga‐fibroblast activation protein inhibitor (FAPI) PET/CT has been proven to be a clear preference in imaging the primary, metastatic, and recurrent tumors across 22 different types of cancers [102, 103, 104]. In particular, ^68^Ga FAPI PET/CT outperforms ^18^F‐FDG PET/CT in identifying the primary lesions for head and neck CUP with more reliable, sensitive, and reproducible imaging modality [103]. Likewise, study from our center verified that ^68^Ga‐FAPI PET/CT improved the detection efficiency of primary lesions in head and neck CUP with negative ^18^F‐FDG PET/CT findings [104].

### 5.4 Histology and IHC staining

IHC analysis on biopsy specimens are required, with histopathological analysis serving as the gold standard for diagnosing CUP before the era of molecular diagnostics [105, 106]. Morphological features of malignancies occasionally offer insights into the tumor type and tissue of origin (ToO). For instance, signet‐ring cancer cells are commonly observed in glandular epithelium of gastrointestinal tract, whereas papillary neoplastic cells are primarily observed in breast and thyroid cancers [107]. However, for poorly differentiated and undifferentiated tumors, relying solely on the morphological analysis is far from sufficient. IHC analysis, based on lineage‐ and organ‐specific markers, was then proposed as a recommendation for classifying the lineage or determining the tumor tissue origin for CUP [21, 108].

Currently, the step‐by‐step process of IHC staining is widely utilized and acknowledged as highly efficient [8]. In the first step, lineage‐specific markers (CK, S100, LCA, and Vimentin) are screened to distinguish carcinoma from lymphoma, sarcoma, and melanoma. For patients with sarcomas of undefined primary, the pathologies are highly variable with histologic heterogeneity [109], which may due to the smallness of the primary tumor to evade detection.

**FIGURE 2:**
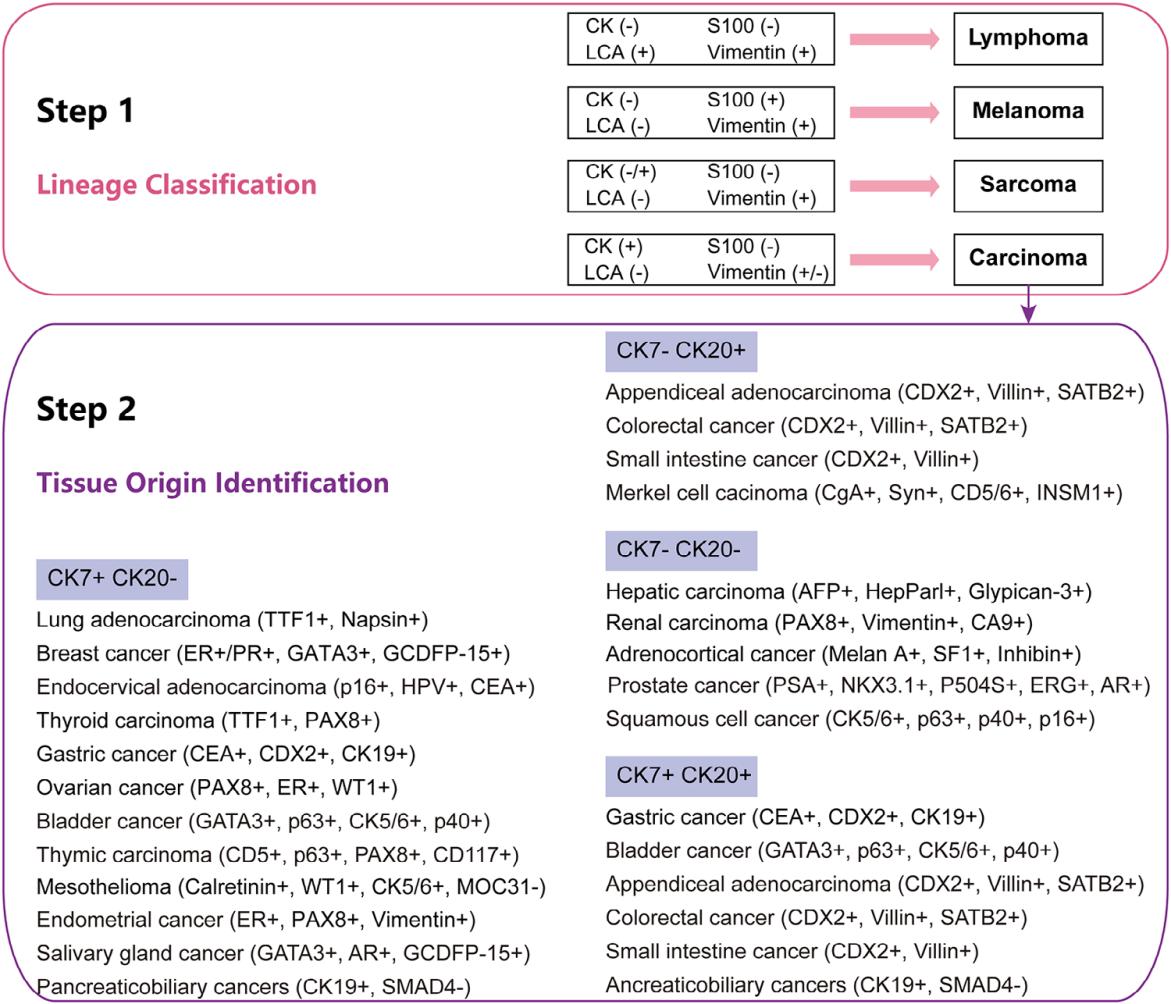
Advisable step‐by‐step IHC screening to identify the tissue origin of CUP. Step one: lineage‐specific markers were recommended to distinguish carcinoma from lymphoma, melanoma, and sarcoma. Step two: organ‐specific markers were applied to identify the tissue origin of CUP.

In the second step, organ‐specific markers are applied to assist in identifying the tissue origin of CUP, which can help narrow down the potential tissue origin of tumors (Figure 2). Once identified as an epithelial malignancy, a combination of CK7 and CK20 is used to speculate on the tissue origin of metastatic tumors [24]. CK20 is predominantly expressed in the urothelial cancer, gastrointestinal tract cancer, and Merkel cell carcinoma, whereas CK7 is primarily negative in colorectal cancer and prostate cancer [110]. Meanwhile, additional markers such as TTF1, p63, GATA3, PSA, β‐HCG, and Hepar1 were applied to infer the primary lesions of CUP [111]. For cancers that are CK20 negative and CK7 positive, the presence of p63 often suggests lung squamous carcinoma, while the presence of TTF1 largely indicates lung adenocarcinoma [112]. IHC analysis demonstrated high accuracy in identifying lesions derived from NSCLC, breast cancer, colorectal cancer, ovarian cancer, and renal cancer. However, the diagnostic accuracy of IHC analysis alone for identifying metastatic tumors originating from gastric cancer, pancreatic cancer, or cholangiocarcinoma still remains relatively low [113]. In fact, previous studies have shown that the overall diagnostic accuracy of multiple IHC markers alone for diagnosing CUP was approximately 77% [25]. However, using more than 10–12 markers does not substantially enhance diagnostic accuracy for CUP. In addition, factors such as tissue antigen instability, tumor heterogeneity, and subjective interpretation of IHC staining by different pathologists have been confirmed as commonly observed factors contributing to decreased accuracy in CUP diagnosis using IHC staining.

### 5.5 Molecular tumor profiling

**FIGURE 3:**
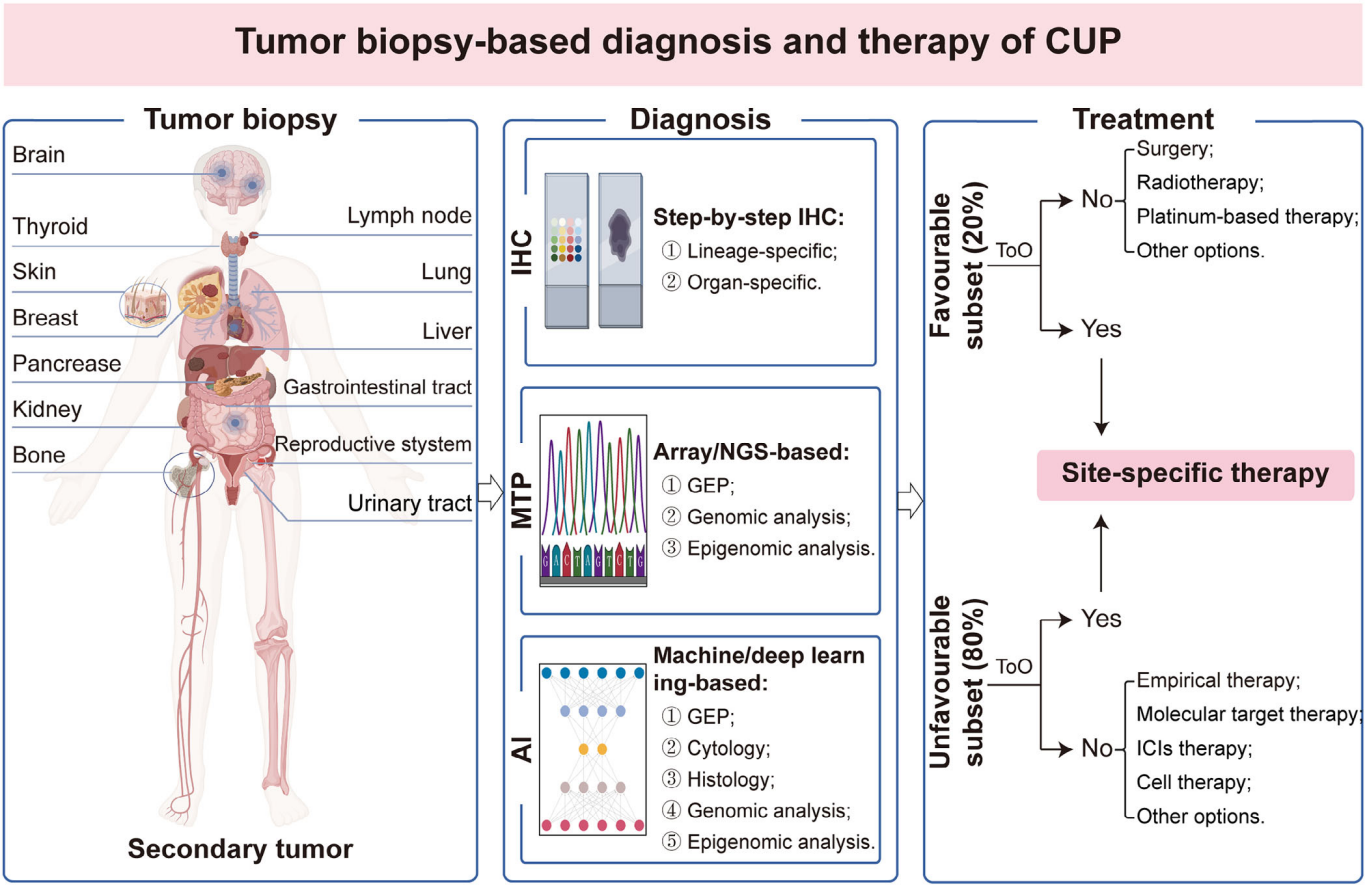
Tumor biopsy‐directed diagnosis and treatment of CUP. In the current era of precision medicine, IHC or MTP‐based methodologies were suggested to assist in identifying the tissue origin of CUP and guiding site‐specific therapy for CUP. *Abbreviations*: AI, artificial Intelligence; GEP, gene expression profiling; IHC, immunohistochemistry; MTP, molecular tumor profiling; NGS, next‐generation sequencing; ToO, tissue of origin.

In the past two decades, significant advancements have been made in tumor molecular diagnostics, resulting in the wide application of MTP as a potential diagnostic method for identifying the tissue origin of CUP [25, 71, 114] (Figure 3). MTP that applied in the diagnosis of CUP includes various methodologies such as GEP by microarray [115, 116] and quantitative reverse transcription PCR (RT‐PCR) [117], as well as the genomic and epigenomic analysis by microarray [27, 36] or NGS [39, 40, 118]. Compared with IHC, MTP requires a smaller amount of tumor tissue and provides higher accuracy in identifying the tissue origin for CUP [25]. MTP has been applied to lay a foundation for guiding site‐specific therapy for CUP due to its outstanding performance in identifying the primary site of CUP [20, 21, 119], refining the management of CUP in the molecular era.

GEP, primarily performed on tumor specimens by RT‐PCR or microarray technologies, involves the detection of gene expression from a panel of genes ranging from 10 to 2000 to aid in identifying the tissue origin of CUP (Table 1). Previous studies have demonstrated that a 10‐gene assay identified a putative tissue origin in 60.6% of cases with CUP [117], while the diagnostic accuracy of the CupPrint^TM^ array that includes 495 tissue‐specific genes can reach up to 92.3% [120]. However, the utilization of expanded gene panels did not enhance the diagnostic accuracy of GEP in assisting with the diagnosis of CUP. Indeed, the ToO Test Kit, comprising 2000 genes [121], demonstrated an accuracy of 76.2% [122]. Gene combination with smaller panels and more robust representation, therefore, is urgently demanded for diagnosing patients with CUP. Furthermore, the CancerTYPE ID assay that contained 92 genes was developed [26] and used for classifying CUP and directing site‐specific therapy for patients with CUP [37]. In fact, the accuracy of the 92‐gene panel has been demonstrated to be over 80% [123]. The ToO Test Kit, which was cleared by the US Food and Drug Administration (US FDA) in March 2018, was one of the two approved assays for identifying the tissue origin of CUP (Table 2). Another one, the Canhelp‐Origin assay, containing 90 specific genes, was cleared by the China National Medical Products Administration (NMPA) in July 2022. Recent studies have revealed the robust performance of the 90‐gene assay in accurately identifying the tissue origin and tumor classification of CUP [85, 124], with overall accuracies ranging from 82.3 to 94.4%, as reported in previous studies [30, 48, 85, 124‐126]. On the whole, the 90‐gene assay demonstrated promising performance, exhibiting higher diagnostic accuracy with a smaller gene panel, which provides a solid foundation for the application of 90‐gene array‐based site‐specific treatment in the clinical therapy of CUP. MicroRNA panels, consisting of 47–89 microRNAs, have been employed to accurately determine the primary site of CUP, exhibiting prediction accuracies ranging from 75 to 95% [127, 128, 129, 130, 131] (Table 1). Overall, data above indicate that GEP exhibits superior performance compared with traditional histopathological techniques in identifying the primary site of CUP, setting a solid foundation for GEP‐guided site‐specific treatment for CUP.

**TABLE 1:** Summary of MTP‐based methodologies applied to identify the tumor tissue origin of CUP and associated prediction accuracy.

Year	Patient (*N*)	Sample	Method	Analyte	Panel	Accuracy	References
2005	13	FF, FFPE	GEP (qRT‐PCR)	RNA	79 genes	84.7%	[132]
2008	38	FFPE	GEP (Microarray)	RNA	495 genes	76.3%	[115]
2008	21	FFPE	GEP (Microarray)	RNA	495 genes	85.7%	[116]
2008	104	FFPE	GEP (qRT‐PCR)	RNA	10 genes	60.6%	[117]
2009	13	FF, FFPE	GEP (Microarray)	RNA	495 genes	92.3%	[120]
2010	20	FFPE	GEP (Microarray)	RNA	495 genes	65.0%	[133]
2010	21	FFPE	GEP (Microarray)	RNA	2000 genes	76.2%	[122]
2011	7	FF	GEP (Microarray)	RNA	2000 genes	NA	[134]
2011	16	FFPE	GEP (Microarray)	microRNA	47 miRNAs	75.0%	[127]
2011	74	FFPE	GEP (qRT‐PCR)	microRNA	48 miRNAs	83.7%	[128]
2012	42	FF, FFPE	Methylation array	DNA	1505 CpGs	77.8%	[135]
2012	52	FFPE	GEP (qRT‐PCR)	microRNA	48 miRNAs	88.5%	[130]
2012	42	FFPE	GEP (qRT‐PCR)	RNA	92 genes	>80%	[123]
2013	24	FFPE	GEP (qRT‐PCR)	RNA	92 genes	75.0%	[25]
2013	252	FFPE	GEP (qRT‐PCR)	RNA	92 genes	NA	[37]
2013	65	FFPE	GEP (Microarray)	RNA	495 genes	81.5%	[136]
2013	192	FFPE	GEP (Microarray)	microRNA	64 miRNAs	85.9%	[131]
2015	49	FFPE	GEP (qRT‐PCR)	RNA	WGS	77.3%	[137]
2016	216	FFPE	Methylation array	DNA	450,000 CpGs	87.0%	[27]
2020	141	FFPE	GEP (qRT‐PCR)	RNA	90 genes	82.3%	[30]
2021	44	FFPE	GEP (qRT‐PCR)	RNA	90 genes	95.4%	[124]
2021	53	FFPE	GEP (qRT‐PCR)	microRNA	89 miRNAs	80–95%	[129]
2022	1417	FFPE	GEP (qRT‐PCR)	RNA	90 genes	94.4%	[85]
2023	68	FFPE	Methylation Sequencing	DNA	WGBS	81–93%	[36]

*Abbreviations*: FF, fresh frozen; FFPE, formalin fixation and paraffin embedding; GEP, gene expression profiling; MTP, molecular tumor profiling; NA, not available; WGBS, whole genome bisulfite sequencing.; WGS, whole genome sequencing.

**TABLE 2:** Products translated from the laboratory to the clinic for diagnosing CUP.

Product	Method	Sample	Analyte	Cancer types	Panel	Accuracy	Clearance	References
CupPrint^TM^	Array	FFPE	RNA	47	495 genes	65.0–92.3%	No	[133]
CancerTYPE ID^®^	qRT‐PCR	FFPE	RNA	50	92 genes	75.0–87.0%	No	[123]
Tissue of Origin Test Kit	Assay	FFPE	RNA	58	2000 genes	76.2–90.8%	Yes (US FDA)	[34]
Canhelp‐Origin^TM^	qRT‐PCR	FFPE	RNA	21	90 genes	82.3–94.4%	Yes (NMPA)	[125]

*Abbreviations*: US FDA, the US Food and Drug Administration; FFPE, formalin fixation and paraffin embedding; NMPA, China National Medical Products Administration.

In addition to GEP, microarray‐ and NGS‐based genomic and epigenomic profiling have also been used to predict the primary site for CUP [39, 71, 137]. Compared with GEP, DNA sequencing shows greater diagnostic value, which may attribute to atypical transcriptional profiles of CUP [138]. Genomic profiling revealed gene mutations, gene rearrangements, and TMB in tumors, allowing for prediction of tumor origin and the guidance of molecularly targeted therapy and ICIs therapy. In addition to genomic profiles, epigenomic alterations, such as DNA methylation (especially decrease in 5‐methylcytosine in tumor cells), have been recognized as a promising target for the development of predictive biomarkers in patients with CUP [27, 118, 135, 139]. Recent studies proved that DNA methylation profile‐based machine learning models successfully predicted the tissue origin in 81–93% of patients with CUP [36, 140‐142]. Importantly, a multicenter, retrospective study revealed that cases of CUP who received DNA methylation microarray‐guided site‐specific first‐line therapy showed obvious improved OS compared with those who received empirical treatment [27, 143]. Furthermore, for tumors where obtaining biopsy specimens is challenging or the sample size is insufficient, cell‐free DNA (cfDNA) methylome sequencing presents a more effective solution to address these issues [144].

Over the past 20 years, scientists have primarily focused on single‐omics research to seek more accurate diagnostic methods for CUP. Recent studies classified CUP based on comprehensive genomic and epigenomic analysis in a prospective observational study and direct personalized therapy for CUP [71]. While we observed the preliminary attempt and great potential of multiomics analysis in primary site identification of CUP, it is essential to acknowledge that this approach often involves higher economic costs and longer time courses for patients. Multiomics analysis in clinical practice may pose challenges unless it significantly improves diagnostic accuracy and remains relatively affordable.

### 5.6 Artificial intelligence

AI have exhibited accurate, reliable, and reproducible performance in cancer diagnosis and therapy guidance [145, 146]. For instance, AI‐based algorithms have been successful used in identifying early lung cancers [147], predicting number of lymph node metastasis in locally advanced gastric cancer [148], skin diseases [149], and CUP [150, 151], conditions that are not easily recognized by human experts. In the field of CUP, machine learning and deep learning models based on GEP [28, 31], genomics [33, 34], epigenomic [36, 152], cytology, and pathology [32, 35] were developed to identify the tumor tissue origin of CUP. The CUP‐AI‐Dx classifier and TOD‐CUP algorithm, both utilizing RNA features, were the first two machine learning models developed for predicting the primary of metastatic cancers. Indeed, the CUP‐AI‐Dx model achieved a top‐1 accuracy of 72.46–98.54% in validation set [28], while the TOD‐CUP algorithm achieved an overall accuracy of 91–96% [31]. However, the performance of both of these two models has not been validated in external CUP cohorts, indicating limited clinical applicability. CUPLR, a genomic profile‐based tumor ToO classifier, achieved an accuracy of over 90% based on cross‐validation and test set with 35 cancer subtypes, successfully determining the primary tumor site for 58% of patients with CUP [34]. In addition, the OncoNPC model, another genomic profile‐based machine learning classifier, illustrated a high level of accuracy in predicting the primary cancer types, achieving a confidence rate of 41.2% across all CUP cases. Significantly, the OncoNPC classifier effectively stratified patients into groups with notable prognostic differences and accurately predicted the survival benefit from OncoNPC‐concordant therapy [33]. Besides, a DNA methylation profile‐based support vector classifier exhibited an excellent performance, with overall accuracies ranging from 81 to 93% in CUP cohort [36], offering potential for clinical decision support in managing CUP.

In addition to molecular features, pathology‐based models were developed when GEP and genomic testing had not yet penetrated clinical practice in low‐resource settings. TOAD, a histology ‐based deep learning model, achieved a top‐1 accuracy of 80% in external validation set and resulted in concordance for 61% of patients with CUP [32]. Histological analysis, in fact, requires less tumor tissue sampling, has a shorter turnaround time, and is more cost‐effective, which may serve as a reliable alternate option for patients without access to MTP. A recent study showed that a cytological histology‐based deep‐learning model (TORCH) predicted the tissue origin in both hydrothorax and ascites with a top‐1 accuracy of 82.6%, which was found to be superior to results obtained by pathologists [35]. Importantly, patients who received TORCH‐guided therapy had better OS than those who did not, highlighting its potential role as a valuable auxiliary method for individualized treatment schemes. In addition to biopsies, the circulating cfDNA methylation‐based machine learning classifier (CUPiD) achieved an agreement of 88.5% with a subsequent or suspected primary tumor diagnosis in patients with CUP [152]. AI enhances diagnostic efficiency in oncology by leveraging machine or deep learning algorithms to analyze large datasets, improve image recognition, and identify patterns in histopathology. By automating routine tasks and providing decision support, AI reduces the time required for diagnosis and minimizes human error. Additionally, AI can optimize resource allocation, leading to cost savings in laboratory and clinical settings. Integrating AI tools into clinical workflows ultimately facilitates early detection and personalized treatment, thereby improving patient outcomes and reducing healthcare expenditures. Researches above indicate that the transcriptomic, genomic, epigenomic characteristics of tumor tissue, as well as pathological and clinical features, play an important role in identifying the primary of CUP.

## 6 Treatment of Patients With Cup

### 6.1 First‐line treatment

Patients with CUP typically present with symptoms and signs of metastatic lesions, leading to a diagnosis at an advanced stage. Surgery, therefore, is only considered applicable to specific subtypes of patients with circumscribed and resectable lesions [2]. Based on previous perspective, patients with CUP were categorized into favorable and unfavorable subgroups with distinct survival according to the clinicopathological criteria for better clinical management [1]. Patients in the favorable group have a median OS of 12 months, whereas those in the unfavorable subgroup only exhibit a median OS of 4 months [63]. Patients in the favorable group receive locoregional treatment or systemic platinum‐based treatment, achieving a similar OS to those of patients with relevant known primary tumors. However, patients in unfavorable subset are typically administered empirical chemotherapy with combination regimens of platinum or taxane. Unfortunately, the responses and survival rates in these cases are generally poor. Site‐specific therapy was therefore widely studied to explore whether it could be superior to empirical therapy in improving patient outcomes.

#### 6.1.1 Empirical chemotherapy

Empirical chemotherapy, containing gemcitabine/platinum or taxane/platinum regimens based on the histological types of CUP, has been recognized as the cornerstone of therapy before site‐specific first‐line therapy is proposed [153, 154, 155]. Indeed, there is no consensus on chemotherapy regimens for CUP in the early stages. After more than 30 years of exploration, empirical chemotherapy regimens containing platinum or taxane achieved an ORR of 10–42% [156, 157], along with a median PFS of 2–6.1 months [158, 159] and a median OS of 6.5–13 months [155, 160], respectively (Table 3).

**TABLE 3:** Summary of clinical efficacy of empirical chemotherapy regimens for CUP.

Year	Chemotherapy regimens	ORR (%)	mPFS (months)	mOS (months)	References
1980	Cyclophosphamide + methotrexate + 5‐fluorouracil (CMF) vs. doxorubicin + mitomycin C (DM)	4.5 vs. 36.0	NA	13 vs. 18	[161]
1986	5‐Fluorouracil + driamycin + mitomycin (FAM)	30	NA	10	[162]
1991	Cisplatin	19	NA	7.5	[163]
1997	Carboplatin + paclitaxel + etoposide	47	NA	13.4	[164, 165]
1998	Carboplatin + epirubicin + etoposide	37	NA	10	[166]
1998	Carboplatin + etoposide (EC)	23	NA	5.6	[167]
1998	Mitomycin C + epirubicin + cisplatin vs. mitomycin C	50 vs. 17	4.5 vs. 2.5	9.4 vs. 5.4	[168]
1999	5‐Fluorouracil + cisplatin (CFTam)	27	NA	4	[169]
2000	Paclitaxel + carboplatin (TC)	38.7	NA	13	[155]
2000	Docetaxel + cisplatin (TC) vs. docetaxel + carboplatin (TP)	26 vs. 22	NA	8 vs. 8	[153]
2001	Etoposide + carboplatin (EC) vs. paclitaxel + 5‐fluorouracil + leucovorin	19 vs. 19	8.4 vs. 6.5	NA	[170]
2001	Paclitaxel + carboplatin (TC)/docetaxel + cisplatin (TP)/docetaxel + carboplatin (TC)	27	NA	10	[154]
2002	Gemcitabine + carboplatin + paclitaxel	25	6	9	[171]
2003	Gemcitabine + cisplatin (GP) vs. irinotecan + cisplatin (IP)	5 vs. 38	NA	8 vs. 6	[158]
2003	Gemcitabine + cisplatin + etoposide	36.6	NA	7.2	[172]
2004	Gemcitabine + docetaxel (GT)	40	2	10	[173]
2004	Paclitaxel + carboplatin + etoposide + gemcitabine + irinotecan	29.7	5.7	9.1	[174]
2004	Etoposide + carboplatin + doxorubicin	26.5	4	9	[159]
2004	Paclitaxel + cisplatin (TP)	42	4	11	[175]
2005	Gemcitabine + irinotecan	10	NA	4.5	[176]
2005	Paclitaxel + carboplatin (TC)	23	4.1	6.5	[156]
2005	Gemcitabine + oxaliplatin (GemOx)	29	3.1	12.8	[177]
2006	Cisplatin + gemcitabine + paclitaxel vs. cisplatin + gemcitabine + vinorelbine	48.5 vs. 42.3	NA	9.6 vs. 13.6	[178]
2008	Docetaxel + carboplatin (TC)	32	5.5	16.2	
2008	Oxaliplatin + irinotecan	13	2.7	9.5	[157]
2009	Paclitaxel + carboplatin + bevacizumab + erlotinib	63.0	8.0	12.6	[179]
2009	Oxaliplatin + capecitabine (XELOX)	11.7	2.5	7.5	[180]
2009	Paclitaxel + carboplatin (TC) vs. gemcitabine + vinorelbine (GN)	23.8 vs. 20.0	6.1 vs. 3.2	11.0 vs. 7.0	[181]
2010	Oxaliplatin + capecitabine (XELOX)	18.8	3.7	9.7	[182]
2012	Cisplatin vs. gemcitabine + cisplatin (GP)	16 vs. 19	3 vs. 5	8 vs. 11	[183]
2015	Belinostat + carboplatin + paclitaxel (BelCaP) vs. carboplatin + paclitaxel (CaP)	45 vs. 21	5.4 vs. 5.3	12.4 vs. 9.1	[184]
2016	Oxaliplatin + leucovorin + 5‐fluorouracil (mFOLFOX6)	35.0	3.0	9.5	[185]

*Abbreviations*: NA, not available; ORR, overall response rate; OS, overall survival; PFS, progression‐free survival.

Since adenocarcinoma is the most common histological type of CUP, the initial treatment approach typically emphasizes the use of regimens specifically designed for adenocarcinoma. Classical combination therapy of monthly circles of cyclophosphamide, methotrexate, and 5‐fluorouracil (CMF), first applied as first‐line chemotherapy for breast cancer [186], was utilized to treat ACUP and achieved a median OS of 13 months. In 1980, Professor Brodie conducted a study comparing the response rate and survival benefit of CMF regimen with a combination therapy of doxorubicin and mitomycin C (DM), and they proved that patients who received the DM therapy experienced a significantly improved median OS of 18 months [161]. In addition, FAM (5‐fluorouracil, adriamycin, and mitomycin) combination regimen was recommended as an empiric therapy for patients with ACUP. In 1986, Professor Schein observed a median OS of 10 months on 45 patients who received the FAM regimen as first‐line therapy [162]. In fact, no significant advantages of FAM regimen have been identified, even though it was prominently evidenced in adenocarcinomas with specific primaries. Subsequently, cisplatin‐based regimens were applied in the clinical treatment of CUP, but the prognosis remained unsatisfactory [163]. Considering the wide application of oxaliplatin‐based therapy in gastrointestinal tumors, the combination of oxaliplatin with capecitabine (XELOX) and the combination of oxaliplatin, leucovorin, and 5‐fluorouracil (mFOLFOX6) have been attempted for CUP, achieving a median PFS of 2.5–3.7 months and a median OS of 7.5–9.7 months, respectively [180, 182, 185].

Since 2000, the paclitaxel plus carboplatin (TC) regimen has been widely used in the treatment of patients with CUP, owing to its demonstrated antitumor effects in various types of cancer. In a phase II study, the ORR was 38.7%, and the median OS was 13 months. Patients with nodal or pleural metastasis, in particular, exhibited a better response rate to TC treatment [155]. From then on, taxane plus platinum therapy emerged as a cornerstone in managing CUP, with no significant difference observed between cisplatin and carboplatin in terms of improving patient outcomes [153, 156, 160, 170, 171, 175, 181, 185]. Further, gemcitabine‐ and platinum‐based regimens have shown promise as alternative treatment options for CUP, with a median OS ranging from 6 to 13.6 months [158, 171, 174, 177, 178, 183]. Despite the application of multiple chemotherapeutic regimens in the clinical management of CUP, there has been no significant improvement in prognosis, which has planted the seeds for the proposal of site‐specific therapy.

#### 6.1.2 Site‐specific therapy

With the advancements in treatment options for certain cancer types, such as ICIs being accepted as the first‐line therapy for metastatic NSCLC [187] and hepatocellular carcinoma [188], site‐specific treatment is anticipated to play a significant role in improving patient outcomes of CUP. In order to guide site‐specific therapy, MTP has been utilized, employing methods such as GEP, as well as genomic and epigenomic profiling by NGS [27, 37–39, 119]. In the last decade, several clinical trials have been conducted globally to investigate and determine the efficacy and safety of site‐specific therapy compared with empirical chemotherapy for CUP (Table 4).

**TABLE 4:** Comparisons of efficacy between empirical chemotherapy and site‐specific therapy in patients with previously untreated CUP.

		Patient (N)		Median PFS (months)		Median OS (months)		
Year	Method	Empiric	Site‐specific	Empiric	Site‐specific	Empiric	Site‐specific	References
2013	GEP	396	194	NA	NA	9.1	12.5	[37]
2016	Microarray	61	31	NA	NA	6.0	13.6	[27]
2018	IHC	34	56	4.2	5.1	10.7	20.3	[119]
2019	GEP	51	50	4.8	5.1	12.5	9.8	[38]
2020	NGS	/	97	/	5.2	/	13.7	[39]
2022	NGS	78	17	NA	NA	14.7	23.6	[40]
2024	GEP	91	91	6.6	9.6	19.0	28.2	[41, 42]

*Abbreviations*: GEP, gene expression profiling; IHC, immunohistochemistry; NGS, next‐generation sequencing; OS, overall survival; PFS, progression‐free survival.

Indeed, among the various MTP, GEP was the first to be used in predicting the tissue origin and providing guidance for site‐specific therapy in patients with CUP. In a prospective clinical trial performed by the Sarah Cannon Research Institute in 2013, a 92‐gene expression profile was used to guide standard site‐specific first‐line therapy for CUP [37]. In this trial, patients who received assay‐directed site‐specific therapy achieved a median OS of 12.5 months, which was superior to the median OS of 9.1 months observed in the historical control group receiving empirical chemotherapy. However, it is essential to acknowledge that comparisons with historical results may introduce potential biases. Therefore, it is crucial to conduct further studies to determine whether site‐specific therapy improves patient outcomes of CUP. Furthermore, findings from a prospective, multicenter, randomized phase II clinical trial conducted in Japan indicated that GEP‐directed site‐specific therapy did not significantly improve the median PFS (5.1 vs. 4.8 months, *p* = 0.550) and OS (9.8 vs. 12.5 months, *p* = 0.896) of CUP compared with empirical paclitaxel and carboplatin therapy [38]. Nevertheless, it is important to note that this research still has certain limitations. These include the small sample size and the limited predictive accuracy of the diagnostic model which was found to be only 78.6%, as applied in this study. However, it is encouraging to note that patients in the favorable subgroup who received GEP‐guided site‐specific therapy achieved better median OS and PFS compared with those in the unfavorable subset, which provides some evidence of the superiority of site‐specific therapy for CUP. In a later study from the same group, researchers conducted a phase II clinical trial to assess the clinical value of NGS‐based site‐specific and targeted therapy in previously untreated CUP within the unfavorable subset [39]. In this study, patients with CUP achieved a high 1‐year survival rate of 53.1%, along with a median PFS of 5.2 months and a median OS of 13.7 months. However, this study did not include a comparator group to evaluate the efficacy of NGS‐based site‐specific and targeted therapy in comparison with empirical therapy. Furthermore, in another retrospective study, patients received IHC‐directed site‐specific therapy showed improved median OS compared with those who received empirical therapy in unfavorable subset (20.3 vs. 10.7 months, *p* = 0.03) [119]. Again, a multicenter, retrospective analysis illustrated that DNA methylation profile (EPICUP)‐based site‐specific therapy significantly improved the median OS of patients with CUP compared with those who received empirical therapy (13.6 vs. 6.0 months, *p* = 0.0051) [27]. However, it is important to note that both of these previously mentioned retrospective clinical trials have small sample sizes, which limits the availability of strong evidence regarding the potential improvement of patient prognosis with site‐specific therapy. To summarize, currently available studies comparing site‐specific therapy with empiric chemotherapy exhibit significant deficiencies and notable limitations, which include study design, patient recruitment criteria, heterogeneity among the CUP classifiers, as well as incomparable therapies. Given the inherent limitations of the present researches and the urgent demand to optimize therapeutic strategies for CUP, further well‐designed studies are needed to establish the status of site‐specific therapy for clinical management of patients with CUP.

To address this challenge, our center designed the pioneering worldwide prospective randomized phase III study, which was known as Fudan CUP‐001, to investigate the clinical efficacy and safety of 90‐gene expression assay‐directed site‐specific therapy compared with empirical chemotherapy [41, 189, 190]. Our study proved that site‐specific therapy contributed to a significantly longer median PFS compared with empirical chemotherapy (9.6 vs. 6.6 months, *p* = 0.017). Furthermore, patients who received site‐specific treatment exhibited a favorable median OS compared with those treated with empirical chemotherapy (28.2 vs. 19.0 months, *p* = 0.099). Taken together, our study represents a milestone to show improved PFS and favorable OS based on GEP‐guided site‐specific treatment, as opposed to empirical chemotherapy, in the first‐line therapy for CUP across the globe to date. This significant breakthrough not only opens a new chapter for the clinical management of CUP, but also establishes site‐specific first‐line treatment as a cornerstone in improving patient prognosis for CUP.

#### 6.1.3 Molecularly guided therapy

ICIs and targeted therapy have improved the prognosis and survival of several cancer types in recent years, especially for patients with advanced NSCLC [191] and hepatocellular carcinoma [188]. In CUP, the median OS for those receiving NGS‐based molecularly guided therapy (MGT) was 23.6 months [40]. However, it remains uncertain whether first‐line MGT can enhance patient outcomes in cases of CUP. To address this challenge, an open‐label, randomized, phase II study called CUPISCO was conducted to compare the efficacy and safety of genomic profiling‐guided therapy versus empirical chemotherapy in patients with newly diagnosed, unfavorable, nonsquamous CUP [3, 192]. In the intention‐to‐treat population, patients in the MGT group achieved a median PFS of 6.1 months, whereas patients in the empirical group achieved a median PFS of 4.4 months (*p* = 0.0079). CUPISCO emphasized the significant importance of including genomic profiling in the initial diagnostic work‐up for patients with newly diagnosed, unfavorable CUP to expand therapy strategies and improve prognosis. In summary, both the CUP‐001 and CUPISCO studies confirmed that precision treatment (GEP‐guided site‐specific therapy or MGT) leads to improved patient outcomes for previously untreated CUP when compared with empirical chemotherapy. However, there are some differences between the two studies in terms of study design, patient recruitment, and treatment regimens. The CUPISCO study no longer emphasizes the importance of identifying the primary tumor; instead, it places greater focus on finding therapeutic targets.

In addition, ICIs‐based therapy is also a promising choice for CUP [193]. Previous studies confirmed that CUP patients treated with ICIs achieved an ORR of 29% [73]. Indeed, studies have validated that 28% of patients with CUP exhibit one or more predictive biomarkers for ICIs [194]. Among them, 14% show high expression of PD‐L1 (TPS > 50%), 1.8% present with MSI‐high and 11.8% have a TMB of 17 or more mutations per megabase [65], which provide a foundation for the future application of combination immunotherapy in patients with CUP. Further, HER2‐targeting ADC may be another promising therapeutic option for patients with CUP, as previous studies have documented that HER2 is overexpressed in 10% of CUP [27, 79], which needs to be further investigated.

### 6.2 Second‐line treatment

From four decades of efforts, amount of clinical trials had been conducted to enhance the patient outcomes of CUP. CUP‐001 establishes the status of site‐specific therapy in first‐line treatment of patients with CUP. However, despite the administration of site‐specific first‐line therapy, the median PFS for CUP remains less than one year. Research on effective second‐line therapy aimed at improving patient outcomes has emerged over the past 20 years (Table 5).

**TABLE 5:** Efficacy of second‐line treatment for patients with previously treated or recurrent CUP.

Year	Regimens	Patient (*N*)	ORR	Median PFS (months)	Median OS (months)	References
2001	Gemcitabine	39	7.7%	5	/	[195]
2005	Gemcitabine + irinotecan	40	10.0%	/	4.5	[176]
2007	Bevacizumab + erlotinib	51	9.8%	3.9	7.4	[196]
2010	Capecitabine + oxaliplatin	25	13.6%	2.3	3.9	[197]
2010	Capecitabine + oxaliplatin	48	18.8%	3.7	9.7	[182]
2022	Nivolumab	45	22.2%	4.0	15.9	[198]
2022	Pembrolizumab	29	20.0%	4.1	11.3	[199]
2023	Nivolumab + ipilimumab	31	15.9%	2.5	3.8	[200]

*Abbreviations*: ORR, overall response rate; OS, overall survival; PFS, progression‐free survival.

In the past chemotherapy‐based therapy eras, gemcitabine and gemcitabine in combination with irinotecan achieved an ORR of 7.7–10.0% in patients with previously treated CUP [176, 195], which showed modest activity for recurrent or refractory CUP. In the later era of targeted therapy, the combination inhibition of vascular endothelial growth factor and EGFR with bevacizumab and erlotinib did not significantly improve patient outcomes, achieving an ORR of only 9.8% [196]. With the recent development of immunotherapy, nivolumab and nivolumab in combination with ipilimumab demonstrated superior efficacy, achieving an ORR of 15.9–22.2%, in comparison with earlier studies [198, 200]. Additionally, pembrolizumab monotherapy demonstrated a similar late benefit in investigator‐assessed ORR of 20.0% for recurrent or refractory CUP [199]. However, it still requires further investigation to determine whether patients with previously treated CUP could derive greater benefits from combination therapy involving chemotherapy, targeted therapy, and immunotherapy, which would be of great interest in future studies of later‐line therapy for CUP.

## 7 Future Perspectives

Indeed, the diagnostic approaches and therapeutic optimization for CUP have experienced five decades of evolution. Following the continuous advancements in tumor diagnosis since 2000, CUP diagnosis has transitioned from the era of IHC to the era of MTP, which lays the foundation of site‐specific first‐line therapy to improve patient outcomes of CUP. Despite the superior accuracy of GEP‐based testing in CUP diagnosis, as well as genomic and epigenomic profile‐based procedures, the standard diagnostic techniques still remain inconclusive. Hot directions for future researches in CUP still focus on how to turn CUP into known primary by revolutionary diagnostic approaches, in order to guide therapeutic optimization. Considered together, we propose here the three core questions in the field of CUP that should be further explored in future studies, as detailed below.

First, in terms of diagnosis of CUP, we have observed excellent results with the highest diagnostic accuracy exceeding 90% of methods based on cytology, histology, GEP, genomic, and epigenomic analysis of CUP. Among various panels of GEP‐based methods, the 90‐gene assay stands out as the most prominent, boasting a diagnostic accuracy of 94.4% [85]. However, the 90‐gene testing has several limitations, including its coverage of only 21 common solid tumors, its inability to differentiate between breast cancer and sweat gland cancer, and its low diagnostic efficiency for squamous cell carcinoma, among others. Further studies will aim to apply smaller panels of tumor specific genes to achieve higher diagnostic accuracy and provide improved guidance for site‐specific treatment. Currently, approaches utilizing deep learning or machine learning algorithms have been developed to apply GEP or DNA methylation profiles in the clinical practice for determining the tumor tissue origin of CUP. In addition, even though AI models based on cytology, histology, genomic and epigenomic profiles of CUP have illustrated excellent performance in clinical diagnosis CUP [32‐36, 152], however, these methodologies are primarily at the research stage and are still far from actual clinical application. Although AI‐based multimodal predictive models have illustrated great potential in tumor diagnosis and treatment decision‐making, the application of these approaches in the diagnosis of CUP still has a long way to go due to the high heterogeneity associated with CUP.

**FIGURE 4:**
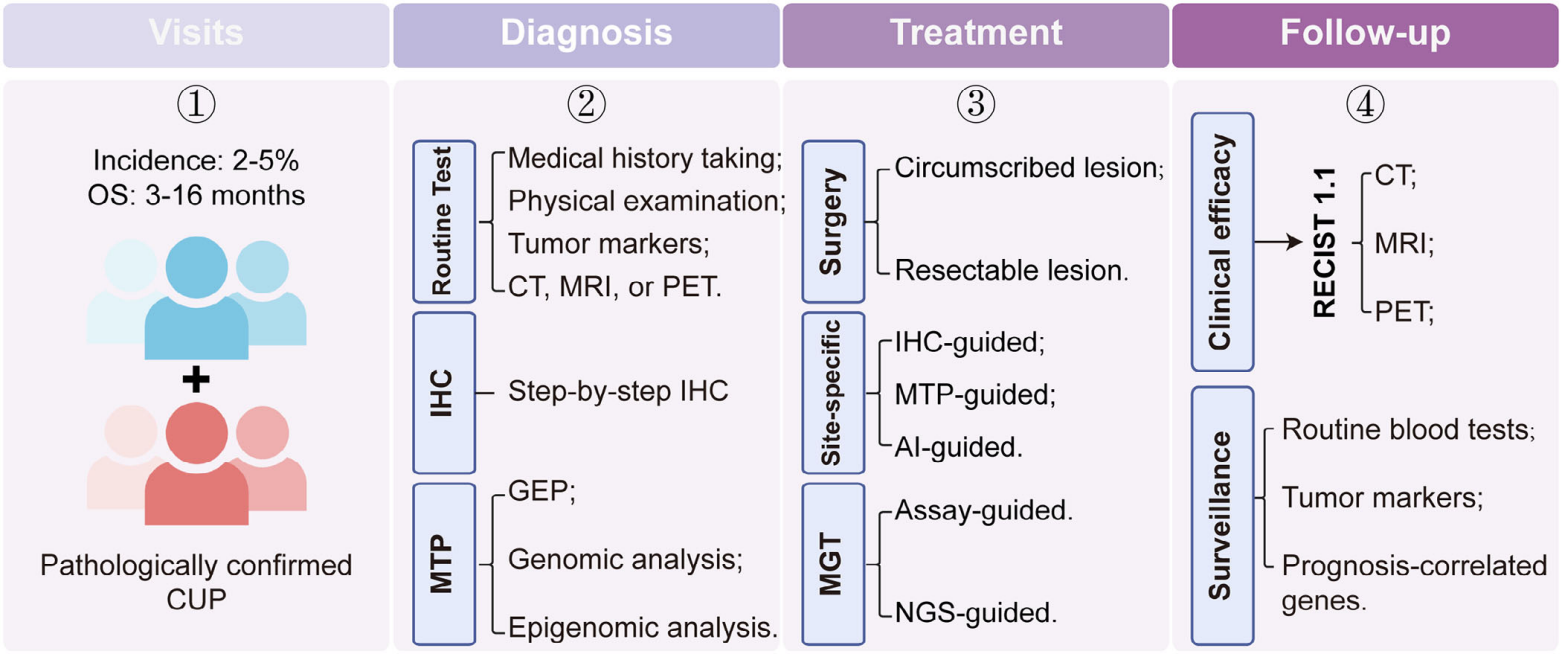
Proposed workflow for the diagnosis and management of CUP. Patients present to the clinic for symptoms and signs of metastatic tumors. A precise diagnosis starts with a comprehensive approach, including routine tests like medical history, physical examination, blood tests, tumor marker detection, imaging examinations, and a step‐by‐step manner immunohistochemistry (IHC) staining. In certain cases, when deemed necessary, molecular tumor profiling (MTP) is recommended. After diagnosis, patients received surgery, site‐specific treatment or MGT according to the clinical and pathologic features. Follow‐up content included response evaluation of antitumoral therapy per the Response Evaluation Criteria In Solid Tumours (RECIST) 1.1 and surveillance guidelines to monitor for recurrence.

Second, it is crucial to shift our perspectives on the relationship between diagnosis and treatment. For patients who can definitely determine the primary site, a standard site‐specific first‐line therapy is recommended as the CUP‐001 documented. However, despite the application of comprehensive examinations, approximately 30% of patients still cannot trace back to the primary site prior to therapy. Most of these cases were histopathologically confirmed as poorly and undifferentiated carcinomas. In this case, palliative care should be prioritized to diagnosis to improve quality of life and prolong OS of patients, especially in the current era of immunotherapy and MGT. As demonstrated by CUPISCO study, even without identifying the primary tumor, patients can achieve good treatment outcomes as long as effective therapeutic targets are identified. Therefore, the question arises: Should we focus on identifying the primary tumor or on finding therapeutic targets? Actually, a few of these patients will progressively present with symptoms and signs associated with the primary tumors as the disease advances. We are therefore currently dedicating our efforts to establish a hierarchical management system for patients based on the histologic, genetic, and clinical features of CUP in the clinical practice, which aims at improving the prognosis and survival of patients with CUP (Figure 4).

Third, the current CUP‐001 and CUPISCO studies confirmed the role of site‐specific therapy and MGT in the first‐line treatment for CUP. Nevertheless, even with the application of site‐specific or MGT first‐line therapy, the median PFS for patients with CUP remains at no more than 1 year. Existing studies have not yet provided effective options for second‐line treatment for patients with CUP after failure of first‐line treatment. Present studies on chemotherapy, targeted therapy, and ICIs‐based treatment, documented an ORR of 7.7–22.2% for previously treated CUP [176, 195, 196, 198‐200]. However, further investigation is needed to determine whether combination therapy involving chemotherapy, targeted therapy, and immunotherapy results in better outcomes for previously treated or recurrent CUP. To address this challenge, our center conducted a prospective, open‐label, single‐arm phase II study to assess the efficacy and safety of combination therapy for patients with CUP who had failed first‐line treatment or experienced disease progression. Findings of this study will be released in the near future and are expected to provide strong evidence for second‐line therapy in CUP.

In summary, we encounter both significant opportunities and challenges in the diagnosis and further‐line treatment of CUP in the current era of precision medicine, and there is still much progress to be made in this field.

## Author Contributions

T. Z., X. W. Z., and Z. G. L. designed the literature, arranged the studies, and drafted the manuscript. X. L., Q. F. W., and X. C. H. prepared the figures and revised the manuscript. All the authors contributed to this manuscript. All authors read and approved the final version of the manuscript.

## Conflicts of Interest

The authors declare no conflicts of interest.

## Ethics Statement

Not applicable.

## Data Availability

Not applicable.
